# Research Landscape of Stem Cell Applications in Musculoskeletal Tissue: A Scoping Review

**DOI:** 10.3390/cells15050456

**Published:** 2026-03-04

**Authors:** Aiyarin Kittilukkana, Puwapong Nimkingratana, Dumnoensun Pruksakorn, Mingkwan Na Takuathung, Nut Koonrungsesomboon

**Affiliations:** 1 Center of Multidisciplinary Technology for Advanced Medicine (CMUTEAM), Faculty of Medicine, Chiang Mai University, Chiang Mai 50200, Thailand; 2 Clinical Research Center for Food and Herbal Product Trials and Development (CR-FAH), Department of Pharmacology, Faculty of Medicine, Chiang Mai University, Chiang Mai 50200, Thailand; 3 Department of Pharmacology, Faculty of Medicine, Chiang Mai University, Chiang Mai 50200, Thailand; 4 Department of Orthopedics, Faculty of Medicine, Chiang Mai University, Chiang Mai 50200, Thailand

**Keywords:** mesenchymal stem cells, regenerative medicine, orthopedics, musculoskeletal diseases, cell-based therapy, scoping review

## Abstract

**Highlights:**

**What are the main findings?**

- Autologous bone marrow-derived MSCs and adipose-derived MSCs remain the predominant cell sources in orthopedic applications, with knee osteoarthritis most frequently used through intra-articular administration.
- The use of perinatal allogeneic MSCs and large-animal models has increased, alongside established small-animal and clinical investigations.

**What is the implication of the main findings?**

- The results help inform the design of more focused, standardized, and clinically relevant trials in orthopedic stem cell research.

**Abstract:**

Stem cell therapy represents an intrinsic part of regenerative medicine, with expanding applications in orthopedic and musculoskeletal research. Although studies span from small-animal models to early-phase clinical trials, the field remains fragmented, with wide variation in stem cell types, delivery methods, and target tissues. A consolidated overview is needed to inform future directions and bridge the gap between preclinical promise and clinical application. This scoping review synthesized evidence from 500 preclinical and clinical studies, identified through systematic searches and screened in accordance with PRISMA-ScR guidelines. Data were extracted on stem cell type and source, delivery approach, targeted tissue and organ, and disease indication. We found that autologous bone marrow-derived mesenchymal stem cells were the most used, with adipose- and perinatal-derived cells gaining prominence in recent years. Small-animal models such as rats and rabbits predominated, while large-animal and human studies focused mainly on knee osteoarthritis. Intra-articular injection was the principal delivery method across both preclinical and clinical settings. By mapping prevailing practices and emerging trends, this review provides a comprehensive reference for researchers, clinicians, and regulatory stakeholders. It highlights translational pathways, identifies critical gaps, and offers evidence to guide the design of safe, effective, and scalable regenerative therapies in orthopedics.

## 1. Introduction

Orthopedic injuries and degeneration, such as osteoarthritis, cartilage degeneration, ligament injuries, and tendon ruptures, are among the leading causes of disability worldwide, especially in aging populations. These conditions impose significant physical, social, and economic burdens and are often associated with chronic pain and reduced quality of life [1,2]. A key challenge in managing musculoskeletal disorders lies in the marked heterogeneity of intrinsic healing capacity among tissues, with bone exhibiting remarkable regenerative capacity, while tendons, ligaments, and cartilage demonstrate poor intrinsic healing capacity [3]. Certain musculoskeletal structures, particularly articular cartilage and the inner regions of the meniscus, exhibit limited vascularization, a well-recognized biological constraint that restricts their intrinsic healing capacity [4]. As a result, these tissues demonstrate limited spontaneous healing following injury [5]. In contrast, other musculoskeletal tissues, such as skeletal muscle, possess abundant vascular supply and a comparatively robust regenerative response [6]. These biological differences partly explain why regenerative strategies, including stem cell-based therapies, have attracted substantial interest across orthopedic and musculoskeletal research. Once damaged, these tissues tend to deteriorate progressively, often culminating in surgical interventions such as joint replacement, which do not restore biological function.

Conventional treatments in orthopedics include anti-inflammatory medications, physical therapy, and surgical procedures that mainly provide symptomatic relief without reversing the underlying tissue degeneration [7]. Cartilage repair techniques such as microfracture or autologous chondrocyte implantation (ACI) offer temporary benefits but frequently result in fibrocartilage formation rather than durable hyaline cartilage [4]. Moreover, tendon and ligament repair surgeries often lead to scar formation and incomplete biomechanical restoration. Despite numerous advancements, these limitations have reinforced the need for regenerative therapies that actively stimulate true tissue repair at a cellular and molecular level.

Stem cell therapy has emerged as a promising solution in regenerative orthopedics due to its potential not only to replace damaged cells but also to modulate the healing environment. Among various cell types, mesenchymal stem cells (MSCs) are the most widely studied for orthopedic applications because of their capacity for multilineage differentiation (e.g., into osteoblasts, chondrocytes, tenocytes) and their potent immunomodulatory effects [8,9]. MSCs can be isolated from diverse tissues such as bone marrow, adipose tissue, synovium, and umbilical cord blood, each offering different advantages in terms of availability, cell yield, and regenerative potential [10]. Moreover, MSCs were believed to regenerate tissues through direct differentiation. Nevertheless, more recent evidence highlights the critical role of their paracrine signaling, including cytokines, growth factors, and extracellular vesicles, which promote angiogenesis, reduce inflammation, and stimulate endogenous repair pathways [11,12]. This dual functionality has made MSCs a focal point in the development of cell-based therapies across various orthopedic tissues. Although MSCs represent the most extensively investigated platform in orthopedic regeneration, the present scoping review was not restricted to MSC-based therapies. Instead, this review intentionally adopted a broad stem cell framework encompassing multiple stem cell classes, including pluripotent stem cell-derived populations and tissue-specific stem/progenitor cells captured within predefined eligibility criteria.

Despite the growth in stem cell research, the field remains highly fragmented. Studies have differed widely in stem cell type, source (e.g., autologous vs. allogeneic), delivery method (e.g., injection, scaffold-based), and anatomical target (e.g., cartilage, tendon, spine), making comparisons challenging and hindering the establishment of best practices [13,14]. The mechanisms underlying MSC-based repair, especially the roles of specific extracellular vesicle contents, microRNAs, and niche interactions, remain incompletely understood. Moreover, while many early-phase trials report short-term benefits, long-term efficacy and safety data are still lacking [15]. There is also considerable variation in regulatory standards and trial design, which complicates clinical translation [16]. These gaps highlight the need for systematic synthesis to clarify what is established, where inconsistencies remain, and which therapies hold translational potential.

Therefore, this scoping review aims to comprehensively map the current research landscape of stem cell applications across musculoskeletal tissue regeneration, including bone, cartilage, tendon, ligament, and skeletal muscle. Drawing from 500 studies, both preclinical and clinical studies, we outline the stem cell type and source, delivery methods, specific musculoskeletal tissues targeted (e.g., bone, cartilage, tendon, ligament, and skeletal muscle), and model used. By distinguishing animal models from clinical studies and analyzing temporal trends, this scoping review highlights prevailing practices, emerging directions, and ongoing gaps.

## 2. Methods

### 2.1. Protocol and Registration

This scoping review followed the Preferred Reporting Items for Systematic Reviews and Meta-Analysis Extension for Scoping Reviews (PRISMA-ScR) guidelines [17]. The protocol was registered on the Open Science Framework (OSF) in accordance with the PRISMA-ScR’s recommendations (Registration DOI: https://osf.io/2k8qd/ accessed on 10 June 2025).

### 2.2. Eligibility Criteria

Eligibility criteria were predefined to ensure consistent study selection. We included original research reporting clinical trials or in vivo animal studies focused on orthopedic or musculoskeletal tissue regeneration (e.g., bone, cartilage, tendon, ligament, or muscle), using stem cells (including but not limited to MSCs) as the primary therapeutic agent delivered via injection or implantation.

We excluded in vitro or ex vivo studies, non-original publications, and interventions involving mixed or undefined cell populations (e.g., SVF, MFAT, or BMAC), cell-free products, endogenous recruitment, co-interventions, or biomaterial-assisted delivery.

A summary of inclusion and exclusion criteria is shown in Table 1.

### 2.3. Information Sources and Search Strategy

To identify relevant studies, we conducted a systematic literature search using four major biomedical databases, including PubMed, Scopus, Embase, and the Cochrane Library. These databases were selected for their coverage of preclinical and clinical research across the fields of medicine and life sciences. Each database spans a wide publication timeframe, ensuring all studies were captured. The initial search was conducted in March 2025, with the last update search in June 2025.

To ensure that our search captured the full spectrum of orthopedic or musculoskeletal-related regenerative applications, we employed a broad targeted keyword strategy. This included terms related to stem cells (e.g., “stem cell”, “mesenchymal stem cell”, “pluripotent stem cell”, “multipotent cell”, “progenitor cell”) and orthopedic tissues and systems (e.g., “bone”, “joint”, “cartilage”, “ligament”, “tendon”, “muscle”, “orthopedic”, “musculoskeletal”). The full electronic search strategy, including Boolean operators and filters used, is available in the Supplementary Materials.

Although our eligibility criteria excluded studies involving non-stem cell populations such as progenitor cells and regenerative cells due to their lack of self-renewal capacity and multipotency characteristic of bona fide stem cells, these terms were intentionally included in the search strategy to ensure comprehensiveness and to minimize the risk of omitting potentially relevant studies. Including these broader terms during the search phase allowed us to capture articles that may reference stem cell interventions but use varying nomenclature or classifications in the title or abstract. Final inclusion was determined through full-text screening based on strict eligibility criteria.

### 2.4. Selection of Sources of Evidence

The selection approach consisted of two stages: (1) title and abstract screening to remove duplicates using SR-Accelerator (https://sr-accelerator.com/) and (2) screening of potentially eligible articles using Rayyan (https://rayyan.ai/ accessed on 20 June 2025) for structured review. Two reviewers independently conducted both screening stages. Any disagreements regarding study eligibility were resolved through discussion and, if necessary, consultation with a third reviewer. Full texts of potentially relevant articles were then retrieved and assessed by the same reviewers, again working independently. Discrepancies in the full-text screening stage were similarly resolved through consensus.

### 2.5. Data Charting and Extraction

A structured data charting and extraction process was performed to systematically capture relevant information from all included studies. A standardized extraction form was developed and piloted to ensure consistency across reviewers. Extracted information included bibliographic details (first author, publication year, article title, journal name), study characteristics (clinical study, animal model, or both), and disease indication (e.g., osteoarthritis, osteonecrosis, tendon injury, rheumatoid arthritis). Specific to stem cell therapies, data were collected on the tissue origin (e.g., bone marrow, adipose tissue, umbilical cord, or synovium), target tissue and organ (e.g., cartilage, bone, tendon; knee, hip, or spine), and the delivery method (e.g., intra-articular, intravenous, or local injection). For studies involving multiple stem cell types, anatomical targets, or delivery strategies, each element was recorded separately to present accurate quantification and mapping of trends in stem cell applications for orthopedic and musculoskeletal tissue regeneration.

A major challenge in synthesizing clinical research is the variability in diagnostic terminology used to describe similar conditions. To enable consistent analysis, disease terms extracted from individual studies were conceptually grouped into broader, clinically relevant categories. This classification was used solely to support data synthesis and interpretation across heterogeneous reports. The standardized human disease categories and their corresponding terms are presented in Figure S1.

The unit of analysis in this scoping review was defined at the publication level, whereby each eligible article was counted once for descriptive analyses. When individual publications reported multiple variables of interest (e.g., different stem cell types, tissue targets, delivery strategies, or experimental models), these variables were extracted and recorded separately to capture methodological heterogeneity. Publications arising from the same clinical trial or experimental cohort, including follow-up reports or secondary analyses, were not merged. Each eligible article was retained as a distinct analytical unit. No assumptions regarding study linkage were imposed in order to maintain methodological transparency. This approach does not influence study counts, as numerical summaries were calculated at the publication level rather than at the extracted-variable level.

When individual publications reported multiple entries within specific parameters (e.g., multiple disease indications, target tissues, or stem cell types), each entry was recorded separately during data extraction. Consequently, a single publication may contribute to more than one analytical category or visualization pathway. Numerical summaries derived from extracted variables therefore represent publication frequencies per parameter rather than unique study counts.

### 2.6. Data Analysis

Data was first tabulated to ensure consistency across key variables, including stem cell type, source, delivery method, target tissues and organs, and animal models. Following the tabulation, visualizations were created to support the findings. The data was then summarized and presented using Microsoft Power BI, R program version 4.4.1, and RStudio (version 2025.05.0+496).

All analyses were conducted descriptively to characterize study designs, stem cell sources, delivery methods, target tissues, and research trends. Given the inclusion of heterogeneous evidence sources, studies were categorized by design type to support context-sensitive interpretation.

## 3. Results

### 3.1. Study Selection

**Figure 1:**
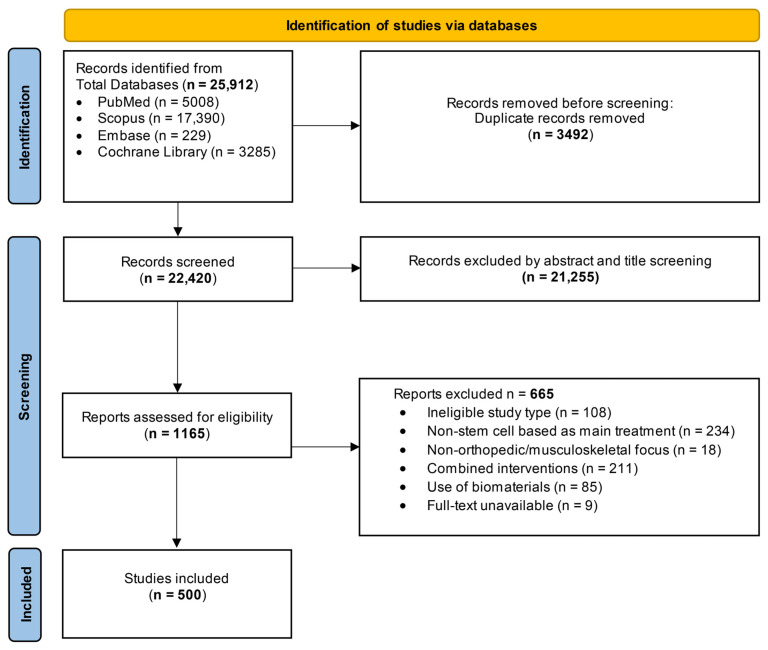
PRISMA 2018 Study Selection Flow Diagram: Preferred Reporting Items for Systematic Reviews and Meta-Analyses (PRISMA) 2018 flow diagram of study selection.

To identify relevant studies for this scoping review, we conducted a comprehensive search across four major databases, PubMed (n = 5008), Scopus (n = 17,390), Embase (n = 229), and the Cochrane Library (n = 3285), resulting in a total of 25,912 records. After removing duplicates, 22,420 records were screened based on titles and abstracts, and 1165 full-text articles were included for eligibility assessment. Ultimately, 500 studies met the inclusion criteria and were incorporated into the final review. The overall screening and selection process is shown in the PRISMA 2018 flow diagram [17], highlighting each step from identification to inclusion (Figure 1).

### 3.2. Preclinical In Vivo Studies of Stem Cell-Based Orthopedic or Musculoskeletal Tissue Regeneration

#### 3.2.1. Stem Cell Source and Type Distribution in Preclinical Animal Models

**Figure 2:**
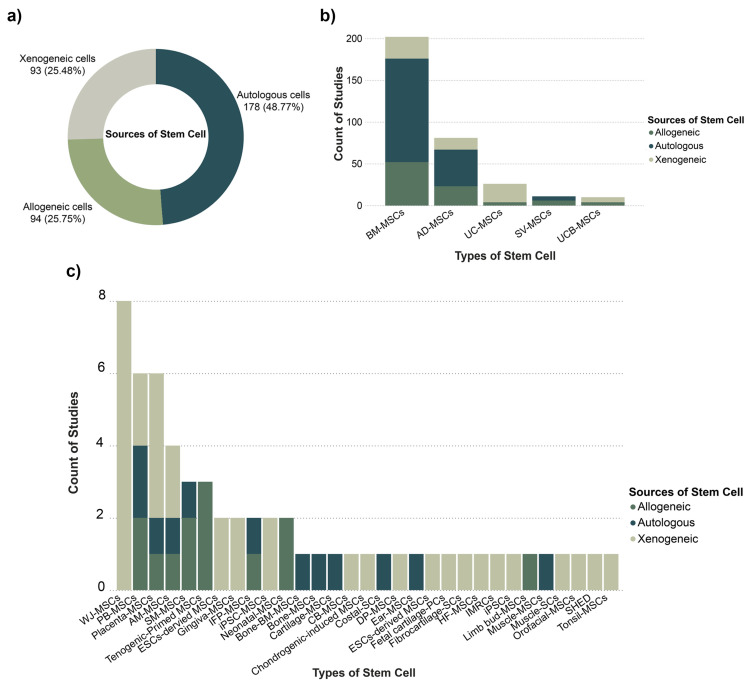
Overview of stem cell types and sources used in in vivo studies. (**a**) Doughnut chart showing the distribution of stem cell sources (autologous, allogeneic, xenogeneic) across in vivo studies. (**b**) Bar chart showing the top five stem cell types stratified by cell source. (**c**) Extended bar chart showing all identified stem cell types, classified by source. Xenogeneic sources emerged as the most commonly employed, following allogeneic and autologous cells. Abbreviations: AD-MSCs, adipose-derived mesenchymal stem cells; AM-MSCs, amniotic membrane-derived mesenchymal stem cells; BM-MSCs, bone marrow-derived mesenchymal stem cells; Bone-BM-MSCs, bone-derived bone marrow mesenchymal stem cells; CB-MSCs, cord blood-derived mesenchymal stem cells; Costal-SCs, costal cartilage-derived stem cells; DP-MSCs, dental pulp-derived mesenchymal stem cells; ESCs-derived MSCs, embryonic stem cell-derived mesenchymal stem cells; Fetal cartilage-PCs, fetal cartilage-derived progenitor cells; Fibrocartilage-SCs, fibrocartilage-derived stem cells; HF-MSCs, hair follicle-derived mesenchymal stem cells; IFP-MSCs, infrapatellar fat pad-derived mesenchymal stem cells; IMRCs, immunity- and matrix-regulatory cells; iPSC-MSCs, induced pluripotent stem cell-derived mesenchymal stem cells; iPSCs, induced pluripotent stem cells; Muscle-SCs, muscle-derived stem cells; PB-MSCs, peripheral blood-derived mesenchymal stem cells; SHED, stem cells from human exfoliated deciduous teeth; SM-MSCs, synovial membrane-derived mesenchymal stem cells; SV-MSCs, synovial-derived mesenchymal stem cells; UCB-MSCs, umbilical cord blood-derived mesenchymal stem cells; UC-MSCs, umbilical cord-derived mesenchymal stem cells; WJ-MSCs, Wharton’s jelly-derived mesenchymal stem cells.

In preclinical animal models, stem cell source selection was largely driven by experimental design considerations, with autologous cells used in nearly half of the studies. The remaining studies employed allogeneic or xenogeneic sources (Figure 2a).

A similar source distribution was observed among the most frequently reported stem cell types (Figure 2b). BM-MSCs were the most studied (n = 202; 100%), predominantly autologous (n = 124; 61%), followed by allogeneic (n = 52; 26%) and xenogeneic use (n = 26; 13%). AD-MSCs ranked second (n = 81; 100%) and were applied across autologous (n = 44; 54%), allogeneic (n = 23; 28%), and xenogeneic models (n = 14; 17%). SV-MSCs were less frequently used but appeared in both autologous (n = 5; 46%) and allogeneic settings (n = 6; 55%).

Perinatal-derived MSCs showed a distinct pattern. UC-MSCs (n = 26; 100%) and UCB-MSCs (n = 10; 100%) were used primarily in xenogeneic (UC-MSCs n = 22; 85%; UCB-MSCs n = 6; 60%) or allogeneic settings (UC-MSCs n = 4; 15%; UCB-MSCs n = 4; 40%), with no reports of autologous use. WJ-MSCs (n = 8; 13%) and placenta-derived MSCs (n = 4; 7%) were used exclusively as xenografts (Figure 2c).

Across the broader set of identified cell types (31 cell types; total n = 60) (Figure 2c), adult tissue-derived cells from cartilage, tendon, bone, and muscle were consistently used autologously. In contrast, peripheral blood-, placenta-, and amniotic membrane-derived MSCs were applied across autologous, allogeneic, and xenogeneic settings, while tenogenic-primed MSCs and neonatal- and limb bud-derived cells were limited to allogeneic use.

Taken together, these findings show that preclinical studies predominantly used autologous adult-tissue MSCs, while perinatal-derived MSCs were mainly applied in xenogeneic or allogeneic settings, and a smaller subset of studies used donor-derived allogeneic products.

#### 3.2.2. Mapping Preclinical Stem Cell Applications: A Sankey Visualization

**Figure 3:**
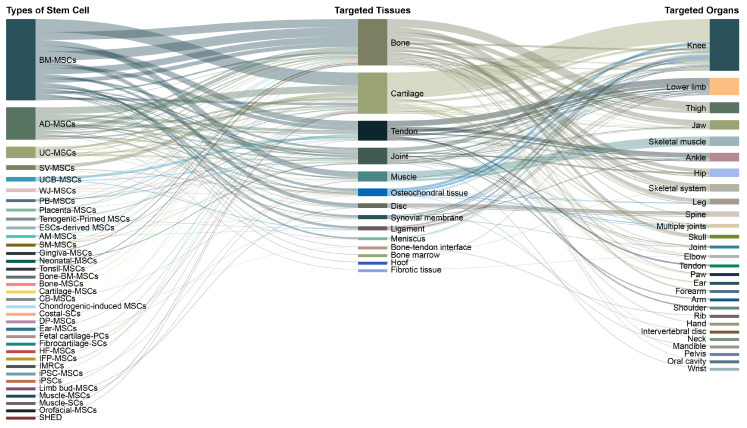
Stem Cell Target Pathways in Preclinical Models: Sankey diagram illustrating the relationships among stem cell types, targeted tissues, and anatomical organs in in vivo studies. BM-MSCs were the most widely used cell type, primarily targeting bone, cartilage, and tendon tissues. The knee was the most frequently addressed organ, followed by the lower limb and the thigh. The figure highlights both the predominance of certain MSC types and the wide variety of anatomical targets studied in preclinical musculoskeletal models. Values represent publication frequencies across the extracted analytical parameters and do not indicate quantitative evidence of efficacy. Abbreviations: AD-MSCs, adipose-derived mesenchymal stem cells; AM-MSCs, amniotic membrane-derived mesenchymal stem cells; BM-MSCs, bone marrow-derived mesenchymal stem cells; Bone-BM-MSCs, bone-derived bone marrow mesenchymal stem cells; CB-MSCs, cord blood-derived mesenchymal stem cells; Costal-SCs, costal cartilage-derived stem cells; DP-MSCs, dental pulp-derived mesenchymal stem cells; ESCs-derived MSCs, embryonic stem cell-derived mesenchymal stem cells; Fetal cartilage-PCs, fetal cartilage-derived progenitor cells; Fibrocartilage-SCs, fibrocartilage-derived stem cells; HF-MSCs, hair follicle-derived mesenchymal stem cells; IFP-MSCs, infrapatellar fat pad-derived mesenchymal stem cells; IMRCs, immunity- and matrix-regulatory cells; iPSC-MSCs, induced pluripotent stem cell-derived mesenchymal stem cells; iPSCs, induced pluripotent stem cells; Limb bud-MSCs, limb bud-derived mesenchymal stem cells; Muscle-SCs, muscle-derived stem cells; PB-MSCs, peripheral blood-derived mesenchymal stem cells; SHED, stem cells from human exfoliated deciduous teeth; SM-MSCs, synovial membrane-derived mesenchymal stem cells; SV-MSCs, synovial-derived mesenchymal stem cells; UCB-MSCs, umbilical cord blood-derived mesenchymal stem cells; UC-MSCs, umbilical cord-derived mesenchymal stem cells; WJ-MSCs, Wharton’s jelly-derived mesenchymal stem cells.

To explore experimental application pathways in preclinical orthopedic/musculoskeletal research, a Sankey diagram was used (Figure 3). BM-MSCs were the predominant cell type (n = 204; 52%), followed by AD-MSCs (n = 80; 20%), while perinatal-derived MSCs contributed a smaller share (e.g., UC-MSCs n = 28; 7%; UCB-MSCs n = 13; 3%; WJ-MSCs n = 9; 2%). Across targets, bone (n = 115; 29%) and cartilage (n = 102; 26%) were most frequently studied, with tendon as the next major category (n = 49; 13%) (Figure 3). At the organ level, the knee was the predominant anatomical focus (n = 128; 33%), followed by the lower limb (n = 43; 11%) and thigh (n = 27; 7%), with remaining sites distributed across multiple anatomical regions. Taken together, the Sankey mapping highlights a dominant pathway centered on BM-MSCs and AD-MSCs targeting cartilage and bone, primarily in knee-focused models.

#### 3.2.3. Overview of Therapeutic Approaches in Animal Models

**Figure 4:**
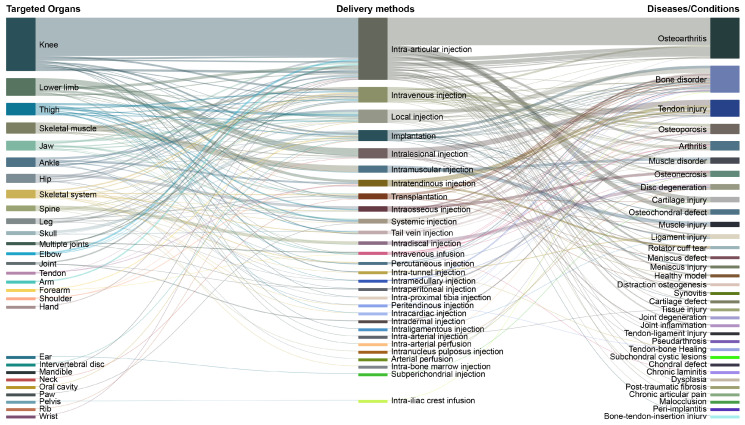
Stem Cell Delivery and Indication Pathways: Sankey diagram showing the associations between targeted organs, delivery methods, and disease/condition indications in stem cell-based orthopedic/musculoskeletal therapies. The left column lists anatomical targets (e.g., knee, lower limb, or spine), the center column shows various MSC delivery methods (e.g., intra-articular injection, intravenous injection, or implantation), and the right column outlines corresponding diseases and conditions (e.g., osteoarthritis, bone disorder, or tendon injury). Flow lines illustrate how specific organs are treated using particular injection or implantation techniques for defined musculoskeletal or joint-related pathologies. Values represent publication frequencies across the extracted analytical parameters and do not indicate quantitative evidence of efficacy.

A Sankey diagram was used to map relationships among target organs, delivery methods, and disease indications in preclinical studies (Figure 4). Osteoarthritis was the most common indication (n = 92; 25%), followed by bone disorders (n = 61; 16%) and tendon injuries (n = 38; 10%). The knee was the most frequently targeted organ (n = 120; 32%).

In terms of delivery, intra-articular injection was the dominant route (n = 140; 38%). Other commonly used approaches included intravenous injection (n = 35; 9%) and local/intralesional injections (general local n = 30; 8%; intralesional n = 23; 6%), with more tissue-specific routes such as intramuscular (n = 16; 4%), intratendinous (n = 15; 4%), and intraosseous injection (n = 12; 3%). Overall, the Sankey mapping indicates that the most prevalent therapeutic pathway in animal models involves intra-articular stem cell delivery to the knee for osteoarthritis.

#### 3.2.4. Translational Mapping of Animal Models, Targeted Organs, and Disease Indications

To examine model selection in preclinical studies, a Sankey diagram mapped recipient species, target organs, and disease indications (Figure S2). Rats were the most common recipient species (n = 131; 37%), followed by rabbits (n = 62; 17%) and mice (n = 50; 14%). Large-animal models contributed a substantial proportion, particularly dogs (n = 41; 12%) and horses (n = 39; 11%). Across indications, osteoarthritis was most frequent (n = 87; 24%), with the knee as the predominant target organ (n = 114; 32%). Bone disorders (n = 57; 16%) and tendon injuries (n = 36; 10%) represented the next major categories, while other conditions comprised smaller fractions.

#### 3.2.5. Mapping Cross-Species Translational Pathways: From Donor to Recipient in Animal Models

To characterize donor–recipient relationships, a Sankey diagram mapped donor species, stem cell types, and recipient models (Figure S3). Rats (n = 100; 27%) and humans (n = 94; 25%) were the most common donor sources, followed by rabbits (n = 52; 14%), horses (n = 42; 11%), and dogs (n = 35; 9%). BM-MSCs dominated across donor sources (n = 195; 52%), followed by AD-MSCs (n = 78; 21%), with other MSC types representing smaller proportions (Figure S3). Recipient species were most commonly rats (n = 141; 37%), rabbits (n = 65; 17%), and mice (n = 55; 15%), with dogs (n = 40; 11%) and horses (n = 39; 10%) representing the main large-animal recipients.

#### 3.2.6. Temporal Trends of Stem Cell Types Used in Preclinical Orthopedic or Musculoskeletal Regeneration

To illustrate the evolving experimental landscape of stem cells used in preclinical orthopedic/musculoskeletal research, we used a heatmap of publication trends from 1998 to 2025 (Figure S4). BM-MSCs remained the most frequently reported cell type across the study period (n = 195; 52%), followed by AD-MSCs (n = 78; 21%). Perinatal-derived MSCs (UC-MSCs, UCB-MSCs, and WJ-MSCs) collectively accounted for 44 studies (12%) and emerged predominantly in later years. In addition, niche or specialized cell types appeared intermittently, indicating diversification of investigated cell sources in recent years.

#### 3.2.7. The Landscape of Disease Targets in Preclinical Orthopedic or Musculoskeletal Research

**Figure 5:**
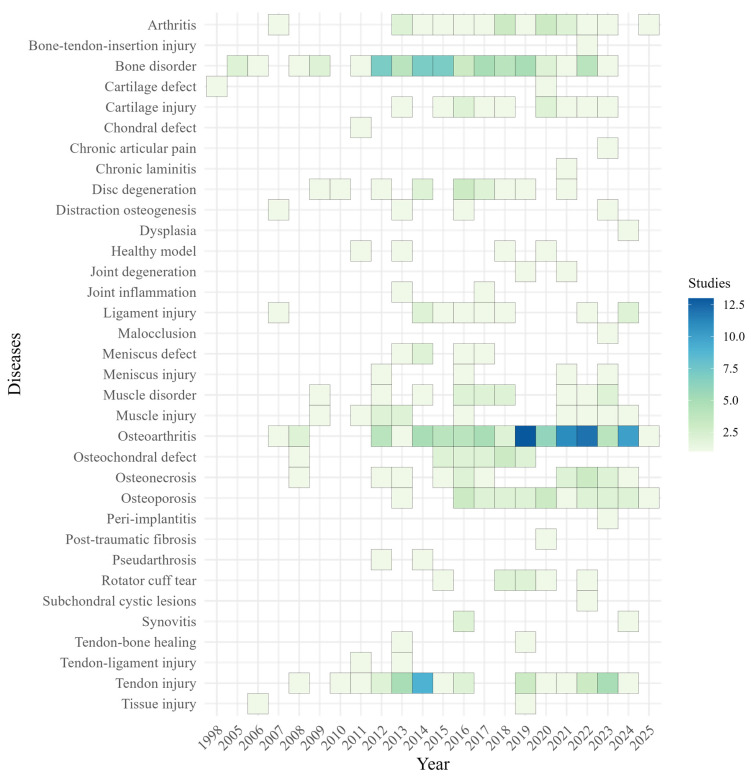
Temporal Trends of Preclinical Disease Targets (1998–2025): Heatmap showing the temporal distribution of orthopedic/musculoskeletal diseases and conditions targeted by stem cell-based therapies in preclinical studies from 1998 to 2025. Rows represent specific diseases or conditions, and columns represent publication years. Color intensity reflects the number of studies published per year for each condition, with darker shades indicating higher frequency. Osteoarthritis, bone disorders, and tendon injuries were the most commonly studied conditions, particularly during 2014–2022, reflecting major research focus areas in orthopedic/musculoskeletal and musculoskeletal regeneration.

To examine shifts in disease targets over time, we generated a heatmap of annual distributions from 1998 to 2025 (Figure 5). Osteoarthritis was the most frequently studied indication (n = 85; 24%), followed by bone disorders (n = 57; 16%) and tendon injuries (n = 36; 10%). Other indications including osteoporosis, arthritis, osteonecrosis, and disc degeneration were consistently represented at lower frequencies, with several niche indications appearing sporadically (Figure 5).

### 3.3. Clinical Assessment of Stem Cell Applications in Orthopedic Tissue Regeneration

#### 3.3.1. Comparison of Autologous and Allogeneic Cell Sources by Type and Frequency

**Figure 6:**
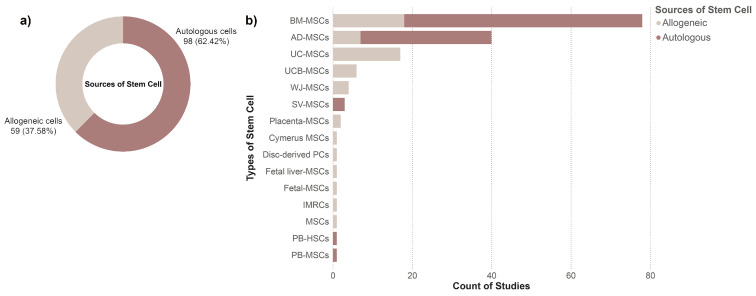
Distribution of stem cell sources in the clinical studies: (**a**) Doughnut chart illustrating the overall proportion of autologous (62%) and allogeneic (38%) stem cell use. (**b**) Stacked bar chart showing the number of studies by stem cell type, further categorized by source (autologous vs. allogeneic). Abbreviations: BM-MSCs, bone marrow-derived mesenchymal stem cells; AD-MSCs, adipose-derived mesenchymal stem cells; UC-MSCs, umbilical cord-derived mesenchymal stem cells; UCB-MSCs, umbilical cord blood-derived mesenchymal stem cells; WJ-MSCs, Wharton’s jelly-derived mesenchymal stem cells; SV-MSCs, synovial-derived mesenchymal stem cells; Placenta-MSCs, placenta-derived mesenchymal stem cells; Disc-derived PCs, progenitor cells; IMRCs, immunity- and matrix-regulatory cells; MSCs, mesenchymal stem cells; PB-HSCs, peripheral blood-derived hematopoietic stem cells; PB-MSCs, peripheral blood-derived mesenchymal stem cells.

To characterize clinical sourcing patterns, we examined the distribution of autologous and allogeneic stem cells across the included studies (Figure 6a). Autologous cells were more frequently used, accounting for 98 studies (62%), whereas allogeneic cells were reported in 59 studies (38%).

When stratified by stem cell type (Figure 6b), BM-MSCs were the most commonly investigated, with most studies employing autologous cells (n = 60; 38%) and a smaller proportion using allogeneic BM-MSCs (n = 18; 11%). AD-MSCs followed a similar distribution, with 33 studies using autologous cells (21%) and 7 using allogeneic cells (4%).

In contrast, perinatal-derived MSCs including UC-MSCs (n = 17; 11%), UCB-MSCs (n = 6; 4%), WJ-MSCs (n = 4; 3%), and placenta-derived MSCs (n = 2; 1%) were reported exclusively in allogeneic applications. SV-MSCs were used only in autologous settings (n = 3; 2%). Other stem cell types were reported infrequently, each appearing in no more than one study.

#### 3.3.2. Mapping Stem Cell Applications in Orthopedic or Musculoskeletal Regeneration: A Sankey Diagram Analysis

To summarize how stem cell therapies are implemented across clinical orthopedic/musculoskeletal indications, a Sankey diagram was generated (Figure S5).

BM-MSCs were the most commonly used cell type (n = 81; 50%), followed by AD-MSCs (n = 40; 25%). Perinatal-derived MSCs represented a smaller but consistent group, led by UC-MSCs (n = 17; 11%). Cartilage was the most frequently targeted tissue (n = 66; 41%), followed by bone (n = 37; 23%) and joint tissue (n = 34; 21%). At the organ level, the knee predominated (n = 97; 60%), with the hip (n = 22; 14%) and spine (n = 9; 6%) representing secondary targets. Overall, the Sankey analysis identified a dominant clinical pathway centered on MSC-based therapies for cartilage and joint conditions of the knee.

#### 3.3.3. Mapping the Clinical Pathway: From Organ to Delivery Method to Disease

**Figure 7:**
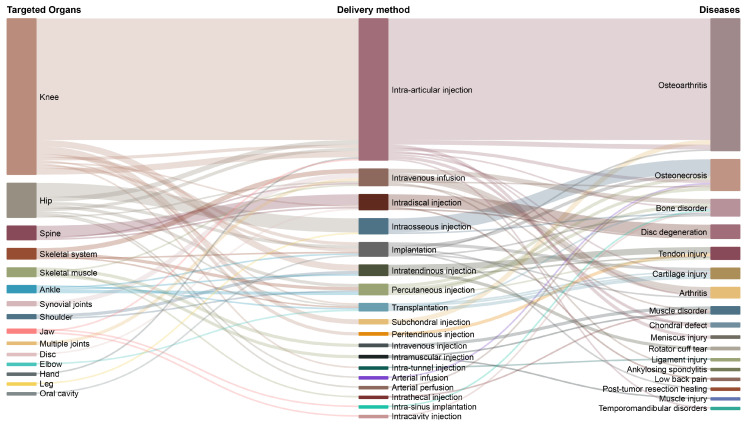
Therapeutic Pathways Linking Organs, Delivery Routes, and Indications: Sankey diagram mapping the therapeutic pathways of stem cell applications by linking targeted organs, delivery methods, and their corresponding clinical indications. Values represent publication frequencies across the extracted analytical parameters and do not indicate quantitative evidence of efficacy.

To examine clinical therapeutic pathways, a Sankey diagram mapped target organs, delivery methods, and disease indications (Figure 7).

The most frequent pathway was knee, intra-articular injection and osteoarthritis, reported in 80 studies (49%). The hip was the second most common target (n = 22; 13%), most often treated via intraosseous injection for osteonecrosis (n = 9; 6%). Spine-related applications primarily involved intradiscal injection for disc degeneration (n = 8; 5%).

Across all studies, intra-articular injection was the predominant delivery route (n = 90; 55%), followed by intravenous infusion (n = 11; 7%) and intraosseous or intradiscal injection (n = 10; 6% each). Other delivery techniques were used less frequently and appeared as isolated applications.

#### 3.3.4. Temporal Trends in the Clinical Use of Different Stem Cell Types

To track the clinical maturation and adoption of stem cell platforms over time, publication trends from 2005 to early 2025 were analyzed using a heatmap (Figure S6). BM-MSCs remained the most frequently reported cell type throughout the study period (n = 77; 49%). AD-MSCs represented the second most common platform (n = 40; 26%), with increased reporting after 2012. Perinatal-derived MSCs, led by UC-MSCs (n = 17; 11%), emerged after 2014 and showed sustained use thereafter. Less frequently reported cell types appeared sporadically, reflecting early-stage or niche clinical investigations. Overall publication activity increased markedly between 2018 and 2023, with peak output in 2020 and 2021 (n = 20; 13% each).

#### 3.3.5. Mapping the Temporal Trends of Clinical Disease Targets

**Figure 8:**
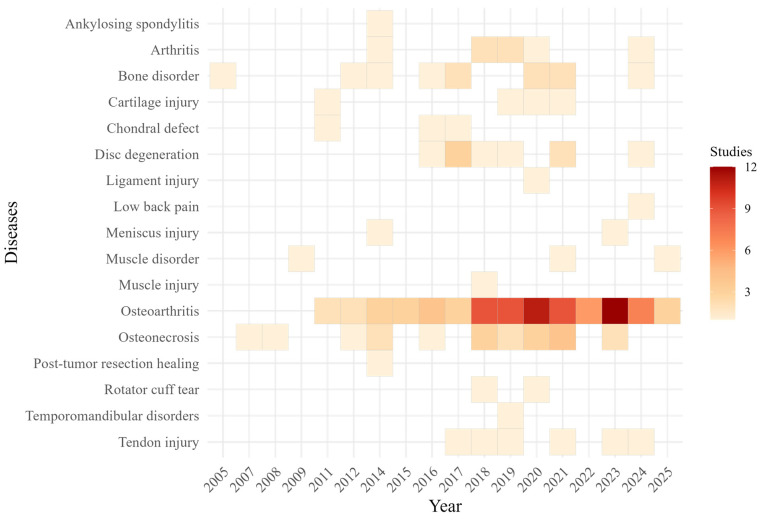
Temporal Trends of Clinical Disease Indications (2005–2025): Heatmap illustrating the annual frequency of clinical studies using stem cells by disease indication from 2005 through early 2025. Darker shades indicate years with higher study counts for each condition. Osteoarthritis is the most frequently studied disease, followed by osteonecrosis, bone disorders, and disc degeneration.

Disease-specific publication trends from 2005 to early 2025 were summarized using a heatmap (Figure 8). Osteoarthritis was the dominant clinical indication, accounting for more than half of all studies (n = 83; 53%). Osteonecrosis was the second most frequently studied condition (n = 20; 13%), followed by bone disorders (n = 11; 7%) and disc degeneration (n = 9; 6%).

Additional indications including tendon injuries, cartilage damage, and other joint-related conditions appeared less frequently and represented smaller fractions of the clinical landscape. Publication activity across disease indications increased substantially after 2018 and remained high through early 2025.

### 3.4. Summary of Key Findings

Across both preclinical and clinical studies, the predominant approach for treating knee osteoarthritis is the intra-articular injection of BM-MSCs and AD-MSCs, as the knee remains the most frequently targeted anatomical site. This consistency underscores the clinical burden of knee joint degeneration and the procedural feasibility of intra-articular delivery.

Autologous stem cells are the preferred cell source in both preclinical and clinical studies, primarily to reduce immunogenic risks. BM-MSCs and AD-MSCs dominate as the most utilized cell types, typically in autologous settings. Clinical studies have increasingly adopted perinatal-derived MSCs such as umbilical cord tissue (UC-MSCs) as allogeneic, off-the-shelf products to meet scalability demands. Preclinical models, meanwhile, frequently incorporate xenogeneic transplantation of human cells to assess translational relevance.

Osteoarthritis continues to be the primary disease focus, but expanding clinical applications now include intraosseous MSC injections for hip osteonecrosis and intradiscal delivery for intervertebral disc degeneration. Notably, a temporal shift in cell sourcing is evident, with the field moving from BM-MSCs to AD-MSCs and perinatal MSCs after 2012–2015, reflecting a broader push toward minimally invasive, scalable therapies.

Preclinical research remains anchored in small animal models, particularly rodents, for mechanistic and safety evaluation. However, there is a discernible transition toward large animal models such as horses and dogs, especially in studies of joint and tendon regeneration, to bridge the translational gap.

Temporal trends indicate shifts in research activity, with peaks observed in both preclinical and clinical publication periods. These patterns reflect evolving investigative focus and growing research interest across experimental and clinical contexts, particularly in knee osteoarthritis.

## 4. Discussion

### 4.1. Preclinical In Vivo Perspectives on Stem Cell Applications in Orthopedic and Musculoskeletal Regeneration

While MSCs dominate the identified literature and consequently shape many observable patterns, the review also captured a heterogeneous subset of non-MSC stem cell platforms. These cell classes differ substantially in developmental origin, differentiation behavior, and translational implications. Therefore, interpretations derived from aggregated mapping should not be viewed as MSC-specific conclusions but rather as reflective of the broader stem cell research landscape.

#### 4.1.1. Strategic Choices in Stem Cell Sources for Animal Studies

Preclinical animal studies remain essential for testing stem cell-based strategies under controlled conditions before clinical translation. A key experimental design decision is the choice of cell source (autologous, allogeneic, or xenogeneic), which directly shapes immune compatibility, feasibility, and translational interpretability. Autologous cells are commonly favored to minimize rejection and reduce the need for immunosuppression, thereby enabling clearer attribution of outcomes to the intervention itself [18]. In contrast, allogeneic approaches facilitate scalable manufacturing and standardized dosing but may require immune modulation or immunosuppressive regimens depending on the model and cell product characteristics [19]. Xenogeneic transplantation, including the use of human cells in rodent models, provides an accessible platform to interrogate human cell behavior in vivo; however, species-specific immune responses and related safety considerations introduce important limitations that constrain direct translational interpretation [20].

#### 4.1.2. The Go-To Stem Cell Types in Orthopedic or Musculoskeletal Research

Across preclinical studies, BM-MSCs and AD-MSCs remained the dominant platforms, reflecting both practical procurement in animal models and their established relevance to musculoskeletal repair [21,22]. AD-MSCs are particularly attractive in translational pipelines because they are readily accessible, expandable, and broadly applied across autologous, allogeneic, and xenogeneic contexts [23,24]. In parallel, the increasing use of perinatal-derived MSCs (e.g., UC-MSCs and WJ-MSCs) signals growing interest in cell sources that can support standardized, off-the-shelf development pathways, although their baseline lineage potency may differ from adult MSCs in ways that matter for specific orthopedic targets [25,26,27,28,29,30].

#### 4.1.3. Mapping Stem Cell Applications: From Tissues to Delivery

Patterns observed in tissue targeting and delivery routes indicate that preclinical study designs often mirror clinically tractable procedures, particularly intra-articular delivery for knee joint disease. This alignment is rational because osteoarthritis and cartilage degeneration represent high-burden clinical problems and intra-articular injection is technically feasible across species [7,31]. Moreover, MSCs are frequently positioned as immunomodulatory and anti-inflammatory agents in joint disease, providing a biological rationale for their repeated evaluation in osteoarthritis models [32,33]. Nevertheless, preclinical delivery choices may also reflect convenience and model availability rather than strict translational optimization, particularly when injection routes or injury models do not adequately reproduce human biomechanics or chronic disease trajectories [34].

#### 4.1.4. Animal Model Selection in Experimental Design

Model choice strongly influences what translation means in a given preclinical study. Rodents dominate because they are cost-effective and well-suited for early mechanistic exploration [35]. Rabbits offer advantages for cartilage repair studies due to joint size and structural features that can approximate certain clinical procedures [36,37]. Mice remain essential for genetic and molecular interrogation of pathways potentially relevant to stem cell function [38,39,40]. Importantly, increased use of large animals such as horses and dogs reflects an effort to bridge biomechanical and immunological gaps, since these species can exhibit orthopedic phenotypes that more closely resemble human conditions [41,42]. However, large-animal work is still relatively limited, and this imbalance has direct consequences for how confidently preclinical results can be generalized to clinical settings.

#### 4.1.5. Evolution of Stem Cell Utilization

Temporal trends suggest progression from foundational BM-MSC research toward more accessible and scalable cell platforms. BM-MSCs initially served as the field’s reference standard based on extensive characterization and musculoskeletal differentiation potential [43,44]. Increased adoption of AD-MSCs after around 2012 likely reflects practical sourcing advantages and a shift toward scalable protocols [23,45]. Rising interest in perinatal-derived MSCs after around 2015 is consistent with broader regenerative medicine goals of standardization and allogeneic product development, building on earlier biological evidence supporting their immunomodulatory and fetal-like properties [25,26,27]. In parallel, expansion of disease targets beyond osteoarthritis and bone defects toward more anatomically complex conditions (e.g., meniscus and rotator cuff pathologies) may reflect a maturing field attempting to address clinically challenging indications [46,47,48,49,50].

#### 4.1.6. Biological Rationale Underlying Observed Preclinical Trends

A key limitation of purely descriptive mapping is that it can obscure why certain platforms dominate and what mechanisms they are implicitly expected to deliver. Across preclinical musculoskeletal models, MSCs are frequently investigated not primarily as direct tissue building blocks, but as biologically active modulators of repair, exerting therapeutic effects largely through paracrine signaling, immune regulation, and trophic support [32,33]. This mechanistic framing helps explain the repeated selection of MSCs in inflammatory degenerative settings such as osteoarthritis, where immunomodulation can be as consequential as structural regeneration [7,31,32,33].

In preclinical in vivo studies, therapeutic outcomes are typically assessed in conjunction with the administered cell dose; however, consistent or linear dose–response relationships are rarely demonstrated. Inconsistencies in reported cell dosages hinder direct comparisons between studies. Variations in dosing units, administration schedules, and reporting methodologies limit the ability to assess dose-dependent effects accurately, underscoring the need for standardized dosing frameworks in orthopedic stem cell research.

Instead, most studies apply doses within a limited range for a given model or indication, reflecting pragmatic experimental choices rather than systematic dose optimization. Consequently, observed efficacy in animal models should be interpreted as context dependent, shaped by both biological mechanism and study design rather than cell dose alone [51].

The frequent use of xenogeneic perinatal MSCs in animal models also has a plausible biological rationale: perinatal-derived MSCs are commonly perceived to have lower immunogenicity and robust secretome-mediated activity, making them attractive for cross-species experiments that aim to test clinically relevant human cells [20,25,26,27]. However, low immunogenicity does not equate to immune invisibility, and xenogeneic settings may still provoke immune-mediated cell clearance or altered host responses that change the apparent effect size [19,20]. As a result, positive outcomes in xenogeneic rodent models may sometimes reflect transient paracrine effects rather than durable engraftment or lineage-driven tissue replacement, which is especially important when interpreting results for bone regeneration or load-bearing repair.

Importantly, biological differences between MSC sources may align with the observed tissue-target patterns. UC-MSCs have been repeatedly described as exhibiting robust chondrogenic differentiation outputs, while demonstrating comparatively limited intrinsic osteogenic potency relative to BM-MSCs [29,30]. As a result, UC-MSC platforms may be more naturally suited to cartilage-leaning indications, unless osteogenic priming or pathway-directed enhancement strategies are applied [28].

Similarly, WJ-MSCs may require osteoinductive conditioning to reach osteogenic performance levels that are more typical for adult MSCs [28]. Together, these mechanistic considerations support the interpretation of preclinical trends. Specifically, the frequent selection of certain MSC sources, particularly perinatal-derived cells, often reflects their immunomodulatory profiles, proliferative capacity, and scalability for allogeneic use rather than inherent lineage suitability for a given target tissue [26,29]. Consistent with this, accumulating comparative studies demonstrate that baseline differentiation potential varies substantially by tissue origin, and may not align with orthopedic lineage demands without additional priming or conditioning [27,28,30]. These distinctions are therefore critical when extrapolating apparent efficacy from animal models to clinical expectations.

#### 4.1.7. Translational Misalignment Between Preclinical Models and Clinical Implementation

Although preclinical mapping highlights dominant pathways (e.g., knee osteoarthritis models treated via intra-articular MSC delivery), the degree of translational alignment varies substantially across study designs [7,31]. First, disease biology differs: many animal models capture acute injury or accelerated degeneration rather than the prolonged, heterogeneous course of human musculoskeletal disease [7,31]. Defect size and tissue scale in rodents may not recapitulate clinically relevant critical-sized defects encountered in human orthopedic disease, particularly in load-bearing tissues. In addition, post-operative loading conditions and joint biomechanics often differ substantially between experimental models and human patients, where non-physiologic loading or short observation windows can mask mechanical failure or overestimate repair durability. Moreover, many preclinical studies rely on young, otherwise healthy animals, whereas clinical populations are typically older and present with comorbidities that shape inflammatory tone, healing capacity, and responsiveness to cell-based therapies. This can inflate apparent efficacy, particularly for therapies that act through short-term immunomodulation [32,33]. Second, immune context differs such that xenogeneic transplantation of human cells into immunocompetent animals can introduce rejection-related confounding [20], whereas immunosuppressed models may overestimate persistence and safety by masking immune-mediated toxicities [19,52]. Third, feasibility and regulatory realism diverge across routes and products. Even for intra-articular injection, arguably the most clinically tractable route, cell retention, dosing volume constraints, and the need for image-guidance or repeat dosing can differ markedly between animal joints and human practice. Some preclinical delivery methods or constructs can be technically feasible in animals but are less scalable or clinically acceptable in routine practice due to procedural invasiveness, imaging requirements, or manufacturing constraints [53]. Likewise, donor-banked or engineered products may appear straightforward in preclinical pipelines but face heightened regulatory expectations for identity, potency, and batch-to-batch consistency in human use [53,54,55].

These gaps underscore why mapping should be paired with critical appraisal: translational value is highest when model choice, immune context, delivery route, and cell product format jointly approximate realistic clinical implementation rather than merely demonstrating proof-of-concept in permissive experimental settings [41,42]. These observations highlight that a substantial proportion of preclinical studies remain exploratory in nature, with variable alignment to realistic clinical and regulatory pathways.

Taken together, these preclinical patterns highlight that the value of this scoping review lies not in reiterating established preferences for specific stem cell sources or models, but in integrating fragmented evidence into a translationally oriented framework. By jointly considering cell source selection, donor–recipient context, delivery routes, and temporal trends, this analysis highlights both areas of convergence and persistent misalignment between experimental design and clinical implementation. This integrated perspective provides context for interpreting apparent efficacy in animal studies and sets the foundation for examining how these preclinical trends translate into real-world clinical practice.

### 4.2. Clinical Translation and Real-World Implementation of Stem Cell Therapies Regenerative Medicine

In contrast to the preclinical mapping above, this section focuses specifically on clinical studies as a distinct translational tier, emphasizing real-world implementation, heterogeneity, and interpretive limitations rather than study frequency. Clinical studies represent the highest translational tier in the evidence landscape but also the area where heterogeneity most directly determines interpretability and real-world relevance [7]. The mapped clinical pathways demonstrate concentration around knee joint disease, especially osteoarthritis [31], yet the clinical meaning of this concentration depends on trial quality, comparators, dosing logic, and follow-up duration rather than frequency alone [56].

#### 4.2.1. Dominance of Autologous Sources and Clinical Implications

The predominance of autologous MSCs in clinical studies likely reflects lower perceived immunogenic risk and more familiar implementation pathways, but it also introduces constraints that directly affect scalability and reproducibility. Autologous therapies require invasive harvesting and are vulnerable to inter-patient variability in cell yield, potency, and proliferative capacity, with downstream implications for product consistency and senescence-associated decline, making standardization challenging [53]. In contrast, allogeneic and perinatal-derived MSCs are positioned as scalable “off-the-shelf” options supported by favorable immunologic profiles [25], but their clinical penetration remains relatively limited [55,57], consistent with ongoing challenges in manufacturing consistency, regulatory complexity, and uncertainties regarding durable efficacy [54].

#### 4.2.2. Therapeutic Focus and Clinical Priorities

Clinical emphasis on cartilage and knee applications is defensible because cartilage has poor intrinsic repair capacity and knee osteoarthritis carries high disease burden and procedural accessibility [7,31]. However, this concentration can also reflect a feasibility funnel, where conditions amenable to intra-articular injection are repeatedly studied while technically complex targets (e.g., load-bearing bone repair with strict biomechanical demands) remain less represented. Accordingly, frequency should not be conflated with clinical readiness across indications.

#### 4.2.3. Therapeutic Pathways in MSC-Based Orthopedic or Musculoskeletal Applications

Knee intra-articular injection remains the dominant clinical pathway, aligning procedural feasibility with a disease area where immunomodulatory benefit could plausibly translate into symptom improvement [7,32,33]. Beyond the knee, several studies have explored organ-specific delivery routes, including intraosseous injection of bone marrow-derived cells into the femoral head for hip osteonecrosis and intradiscal cell injection for intervertebral disc degeneration [58,59]. In this context, comparative evidence across delivery routes remains limited, as route-specific risk and efficacy profiles may differ across tissues and disease mechanisms.

#### 4.2.4. Temporal Trends and Research Evolution

Temporal patterns indicate sustained reliance on BM-MSCs with increasing adoption of AD-MSCs and gradual emergence of perinatal MSC platforms, mirroring a broader shift toward accessibility and scalability [23,24,25,26,27,45]. At the same time, evidence synthesis suggests that clinical benefit for knee osteoarthritis remains uncertain and may be modest, with low-certainty evidence for improvements in pain and function and unclear effects on broader outcomes [56]. This reinforces the need to interpret growth in publication volume as an indicator of interest and feasibility rather than definitive therapeutic maturation [54,55,56].

Across clinical studies, both reported outcomes and dosing regimens vary widely across indications and trial designs. Dosing strategies are commonly adapted to specific disease contexts rather than standardized across conditions, and observed clinical effects should therefore be interpreted as indication specific rather than reflective of generalized dose-dependent effectiveness [60].

### 4.3. Limitations, Critical Gaps and Future Considerations

While approaches such as intra-articular injection for knee osteoarthritis are now well established, there is a lack of adequately powered, head-to-head trials comparing delivery routes, cell sources, dosing strategies, and outcomes across different disease indications. Although some comparative data exist, they remained limited in number and scope and were not comprehensively captured in this scoping review. In the absence of such evidence, it remained challenging to evaluate whether certain interventions offer superior clinical benefit or are primarily adopted based on procedural familiarity or feasibility.

In addition, this scoping review did not stratify included studies by phase of development or evidence maturity (e.g., exploratory preclinical studies versus late preclinical or clinical trials). Instead, studies across different developmental stages were intentionally mapped together to provide a broad overview of the research landscape. While this approach aligns with the primary objective of a scoping review, the absence of phase-based stratification limits the ability to interpret the relative maturity, robustness, or clinical readiness of specific stem cell platforms or therapeutic strategies. This limitation has therefore been acknowledged, as the findings should be interpreted as descriptive of research trends rather than indicative of comparative efficacy or translational strength. Moreover, despite the theoretical advantages of perinatal and allogeneic stem cell sources, including scalability and immune tolerance, their limited clinical uptake underscores ongoing regulatory, logistical, and safety-related barriers. Addressing these challenges will require not only stronger clinical evidence but also harmonized regulatory pathways to enable wider adoption and integration into standard care.

A notable limitation of this review is that the study was not designed to extract or synthesize stem cell dosages and clinical outcomes. While we recognize their importance for comparative effectiveness, including them would have required a level of stratification that falls outside our descriptive scope. Furthermore, the inconsistent reporting of doses across studies varying in units, schedules, and methods makes any meaningful cross-study comparison challenging. These variables may better be suited in future systematic reviews and meta-analyses that focus specifically on dose–response patterns.

In this review, scaffold-based or 3D-construct-assisted delivery systems were excluded to avoid confounding effects, as such constructs may influence cell behavior and outcomes independently. Only studies evaluating stem cells as the primary therapeutic agent were included. The decision to exclude biomaterials, scaffolds, hydrogels, and cell-free products (e.g., extracellular vesicles) was intentional and driven by the aim to specifically examine stem cells as therapeutic entities. Different MSC types exhibit distinct biological behaviors, paracrine profiles, and regenerative capacities, and inclusion of biomaterial-based or combinatorial strategies would introduce substantial heterogeneity related to material composition, mechanical properties, delivery systems, and bioengineering design. Such complexity would substantially limit cross-study comparability and reduce the interpretability of high-level mapping across cell sources, indications, and delivery routes within the scope of this review.

We therefore acknowledge that the findings presented here represent a focused subset of contemporary regenerative strategies and may not fully capture the real-world clinical development in orthopedic tissue engineering, where cell material and cell-free approaches are increasingly explored. This limitation has now been more explicitly stated to clarify the scope and interpretive boundaries of the present analysis. However, the exclusion of 3D systems highlights a critical area for future research, given their potential to enhance cell viability, retention, and regenerative efficacy. Moreover, it remains challenging to determine whether observed benefits are directly from the stem cells or paracrine, immune, or structural effects. Future studies should further explore these variables to clarify the mechanisms driving therapeutic outcomes in stem cell-based orthopedic treatments.

By integrating preclinical and clinical evidence, this review emphasizes stem cells not merely as therapeutic tools but as dynamic biological entities whose source, immunologic context, and delivery route critically influence regenerative outcomes. Taken together, the clinical impact of current MSC-based orthopedic therapies should be interpreted cautiously, as existing trials are heterogeneous, often underpowered, and not designed for definitive comparative effectiveness.

## 5. Conclusions

This scoping review summarizes current stem cell applications in orthopedic and musculoskeletal regeneration across both preclinical and clinical studies. From the 500 studies examined, autologous BM-MSCs and AD-MSCs consistently appeared as the main therapeutic cell types, most often delivered intra-articularly for joint disorders. These patterns reflect practical strengths such as immune compatibility, procedural accessibility, and established clinical familiarity.

The field is gradually shifting. Perinatal and allogeneic cell sources are increasingly being used, large-animal models are being used more frequently to mirror clinical conditions, and research is expanding to a wider range of tissues and pathologies. Despite this progress, notable gaps remain, particularly the scarcity of comparative studies that evaluate different cell types, delivery routes, or indication-specific approaches.

By outlining these trends and gaps, this review offers structured landscape to inform the design of future studies that are more focused, consistent, and clinically relevant, supporting the progression of stem cell therapies toward safe and scalable use in orthopedic and musculoskeletal care.

## Figures and Tables

**Table 1:** Summary of Inclusion and Exclusion Criteria.

Category	Included	Excluded
Study Type	Clinical trial in ○ Experimental study ○ Observational study Preclinical study in animals	In vitro or ex vivo studies Literature reviews Systematic reviews Editorials, commentaries, opinion pieces Modeling studies Case series Case reports
Stem cell-based treatment	Isolated stem cell-based treatment	Studies using mixed cell populations or heterogeneous cell/tissue preparations (e.g., SVF, MFAT, or BMAC) Studies using cell-free products (e.g., exosomes or extracellular vesicles) Endogenous stem cell recruitment
Tissue Target	Orthopedic or musculoskeletal-related tissue regeneration, including but not limited to bone, cartilage, tendon, ligament, joint, and muscle	Studies that did not focus on orthopedic or musculoskeletal tissues
Intervention	Direct therapeutic use of stem cells (e.g., injection, implantation)	Combined interventions where stem cells were not the primary treatment or focus of efficacy (e.g., surgery, biomolecule co-treatment)
Biomaterials	Not applicable	Studies using biomaterials, including: Hydrogels (e.g., alginate, gelatin, fibrin, collagen, hyaluronic acid, or PEG) Scaffolds (e.g., PLGA, PCL, chitosan, or silk fibroin) dECM Microspheres, microcarriers Nanomaterials (e.g., nanofibers)

## Data Availability

The raw data supporting the conclusions of this article will be made available by the authors upon request.

## Supplementary Materials

The following supporting information can be downloaded at: https://www.mdpi.com/article/10.3390/cells15050456/s1, Figure S1. Mapping of Clinical Disease Categories. Figure S2. Mapping of Preclinical Models and Disease Targets. Figure S3. Distribution of Donor Species and Recipient Models. Figure S4. Temporal Trends of Preclinical Stem Cell Types. Figure S5. Stem Cell Pathways Across Tissues and Organs. Figure S6. Temporal Trends of Clinical Stem Cell Types.

## List of Abbreviations

AD-MSCs	Adipose tissue-derived mesenchymal stem cells
AM-MSCs	Amniotic membrane-derived mesenchymal stem cells
BM-MSCs	Bone marrow-derived mesenchymal stem cells
Bone-BM-MSCs	Bone marrow-derived mesenchymal stem cells (from bone)
Bone-MSCs	Bone-derived mesenchymal stem cells
Cartilage-MSCs	Cartilage-derived mesenchymal stem cells
CB-MSCs	Cord blood-derived mesenchymal stem cells
Chondrogenic-induced MSCs	Chondrogenic-induced mesenchymal stem cells
Costal-SCs	Costal stem cells
DDFT	Deep digital flexor tendon
Disc-derived PCs	Disc-derived progenitor cells
DP-MSCs	Dental pulp-derived mesenchymal stem cells
Ear-MSCs	Ear-derived mesenchymal stem cells
ESCs-derived MSCs	Embryonic stem cell-derived mesenchymal stem cells
Fetal cartilage-PCs	Fetal cartilage progenitor cells
Fetal liver-MSCs	Fetal liver mesenchymal stem cells
Fetal-MSCs	Fetal mesenchymal stem cells
Fibrocartilage-SCs	Fibrocartilage stem cells
Gingiva-MSCs	Gingiva-derived mesenchymal stem cells
HF-MSCs	Hair follicle-derived mesenchymal stem cells
IFP-MSCs	Infrapatellar fat pad-derived mesenchymal stem cells
IMRCs	Immunity-and-matrix-regulatory cells
iPSC-MSCs	Induced pluripotent stem cell-derived mesenchymal stem cells
iPSCs	Induced pluripotent stem cells
Limb bud-MSCs	Limb bud-derived mesenchymal stem cells
MSCs	Mesenchymal stem cells
Muscle-SCs	Muscle stem cells
Neonatal-MSCs	Neonatal mesenchymal stem cells
Orofacial-MSCs	Orofacial-derived mesenchymal stem cells
PB-HSCs	Peripheral blood-derived hematopoietic stem cells
PB-MSCs	Peripheral blood-derived mesenchymal stem cells
Placenta-MSCs	Placenta-derived mesenchymal stem cells
SDFT	Superficial digital flexor tendon
SHED	Stem cells from human exfoliated deciduous teeth
SM-MSCs	Synovial membrane-derived mesenchymal stem cells
SV-MSCs	Synovial-derived mesenchymal stem cells
Tenogenic-Primed MSCs	Tenogenic-primed mesenchymal stem cells
Tonsil-MSCs	Tonsil-derived mesenchymal stem cells
UCB-MSCs	Umbilical cord blood-derived mesenchymal stem cells
UC-MSCs	Umbilical cord-derived mesenchymal stem cells
WJ-MSCs	Wharton’s jelly-derived mesenchymal stem cells
