# Supplementary material for: Research Landscape of Stem Cell Applications in Musculoskeletal Tissue: A Scoping Review
Source: Cells. 2026 Mar 4;15(5):456. doi: 10.3390/cells15050456 (PMC12984655; doi:10.3390/cells15050456)
Supplement: Supplementary file 1 [file cells-15-00456-s001.zip › cells-4172187-supplementary.pdf]

Supplementary

Figure S1. Mapping of Clinical Disease Categories

Figure S2. Mapping of Preclinical Models and Disease Targets

Figure S3. Distribution of Donor Species and Recipient Models

Figure S4. Temporal Trends of Preclinical Stem Cell Types

Figure S5. Stem Cell Pathways Across Tissues and Organs

Figure S6. Temporal Trends of Clinical Stem Cell Types

Supplementary

1. Search Strategies

PUBMED

(("stem cell"[Title/Abstract] OR "mesenchymal stem cell"[Title/Abstract] OR "multipotent cell"[Title/Abstract] OR "pluripotent stem cell"[Title/Abstract] OR "progenitor cell"[Title/Abstract])) AND (("bone"[Title/Abstract] OR "joint"[Title/Abstract] OR "cartilage"[Title/Abstract] OR "ligament"[Title/Abstract] OR "tendon"[Title/Abstract] OR "muscle"[Title/Abstract] OR "orthopedic"[Title/Abstract] OR "musculoskeletal"[Title/Abstract])) AND (("clinical trials") OR ("clinical trial") OR ("clinical study") OR ("clinical studies") OR ("animal study") OR ("animal studies"))

SCOPUS

(TITLE-ABS-KEY (("stem cell") OR ("mesenchymal stem cell") OR ("multipotent stem Cell") OR ("pluripotent stem cell") OR ("progenitor cell") OR ("embryonic stem cell") OR ("adult stem cell") OR ("mesenchymal stem cell") OR ("stem cells") OR ("progenitor cells") OR ("multipotent stem Cells") OR ("pluripotent stem cells") OR ("progenitor cell")) AND TITLE-ABS-KEY (("bone") OR ("joint") OR ("cartilage") OR ("ligament") OR ("tendon") OR ("muscle") OR ("orthopedic") OR ("musculoskeletal")) AND TITLE-ABS-KEY (("Clinical trial") OR ("Clinical trials") OR ("Clinical study") OR ("Clinical studies") OR ("animal study") OR ("animal studies")) AND NOT TITLE-ABS-KEY (("in vitro") OR ("review article") OR ("meta-analysis") OR ("systematic review")) AND NOT TITLE-ABS-KEY (("review article") OR ("meta-analysis") OR ("systematic review") OR ("reviews") OR ("review type")))

EMBASE

('stem cell'/exp OR 'stem cell') AND 'orthopedic' AND ('stem cell':ti,ab,kw OR 'multipotent stem cell':ti,ab,kw OR 'pluripotent stem cell':ti,ab,kw OR 'embryonic stem cell':ti,ab,kw OR 'adult stem cell':ti,ab,kw OR 'mesenchymal stem cell':ti,ab,kw OR 'stem cells':ti,ab,kw OR 'progenitor cells':ti,ab,kw OR 'multipotent stem cells':ti,ab,kw OR 'pluripotent stem cells':ti,ab,kw OR 'progenitor cell':ti,ab,kw) AND (bone:ti,ab,kw OR joint:ti,ab,kw OR cartilage:ti,ab,kw OR tendon:ti,ab,kw OR ligament:ti,ab,kw OR muscle:ti,ab,kw OR orthopedic:ti,ab,kw OR musculoskeletal:ti,ab,kw) AND ('clinical trial':ti,ab,kw OR 'clinical trials':ti,ab,kw OR 'clinical study':ti,ab,kw OR 'clinical studies':ti,ab,kw OR 'animal study':ti,ab,kw OR 'animal studies':ti,ab,kw) NOT ('in vitro':ti,ab,kw OR 'review article':ti,ab,kw OR 'meta-analysis':ti,ab,kw OR 'systematic review':ti,ab,kw)

Cochrane

("stem cell") OR ("mesenchymal stem cell") OR ("multipotent stem Cell") OR ("pluripotent stem cell") OR ("progenitor cell") OR ("embryonic stem cell") OR ("adult stem cell") OR ("mesenchymal stem cell") OR ("stem cells") OR ("progenitor cells") OR ("multipotent stem Cells") OR ("pluripotent stem cells") OR ("progenitor cell") in Title Abstract Keyword AND ("bone") OR ("joint") OR ("cartilage") OR ("ligament") OR ("tendon") OR ("muscle") OR ("orthopedic") OR ("musculoskeletal") in Title Abstract Keyword NOT ("in vitro") OR ("exosome") OR ("exosomes") OR ("secretome") OR ("secretomes") OR ("gene therapy") OR ("review article") OR ("meta-analysis") OR ("systematic review") in Title Abstract Keyword NOT ("review article") OR ("meta-analysis") OR ("systematic review") OR ("reviews") OR ("review type") in Publication Type AND clinical OR animal in Title Abstract Keyword - in Trials

2. Standardized Terminology for Disease Classification

To address inconsistencies in diagnostic terminology across studies, disease terms were consolidated into standardized categories to facilitate clearer trend analysis (Figure S1). The most frequently reported condition was osteonecrosis (n = 20; 33.3%), comprising 19 studies (31.7%) using the term “osteonecrosis” and one specifying “post-traumatic osteonecrosis” (1.7%). The bone disorder category (n = 11; 18.3%) encompassed a range of rare skeletal conditions, including “osteogenesis imperfecta” (n = 4; 6.7%), “bone non-union” (n = 2; 3.3%), and single studies on “bone defect”, “post-surgical mandibular lesion”, “delayed union”, “juvenile osteochondritis dissecans”, and “alveolar bone defect”.

Disc degeneration (n = 9; 15.0%) was formed by combining studies on “degenerative disc disease” (n = 6; 10.0%), “intervertebral disc degeneration” (n = 2; 3.3%), and one study using the general term “disc degeneration.”

The arthritis category (n = 7; 11.7%) included “rheumatoid arthritis” (n = 5; 8.3%), “juvenile idiopathic arthritis” (n = 1; 1.7%), and “ankle arthritis” (n = 1; 1.7%).

Three additional categories were used to group tissue-specific injuries. Tendon injury (n = 6; 10.0%) included studies on “tendinopathy” (n = 4; 6.7%) and “Achilles tendinopathy” (n = 2; 3.3%). Cartilage injury (n = 4; 6.7%) included three studies on “cartilage defects” and one on “cartilage injury”. Muscle disorder (n = 3; 5.0%) consisted of two studies on “muscular dystrophy” and one on “Duchenne muscular dystrophy”.

Finally, three smaller categories were created to organize injuries to specific tissue types. Tendon Injury (n = 6; 10.0%) comprised studies on "tendinopathy" (n = 4; 6.7%) and "Achilles tendinopathy" (n = 2; 3.3%). The cartilage injury group (n = 4; 6.7%) brought together three studies on "cartilage defect" with one on "cartilage injury." Lastly, Muscle Disorder (n = 3; 5.0%) included

two studies on "muscular dystrophy" and one on the more specific "Duchenne muscular dystrophy".

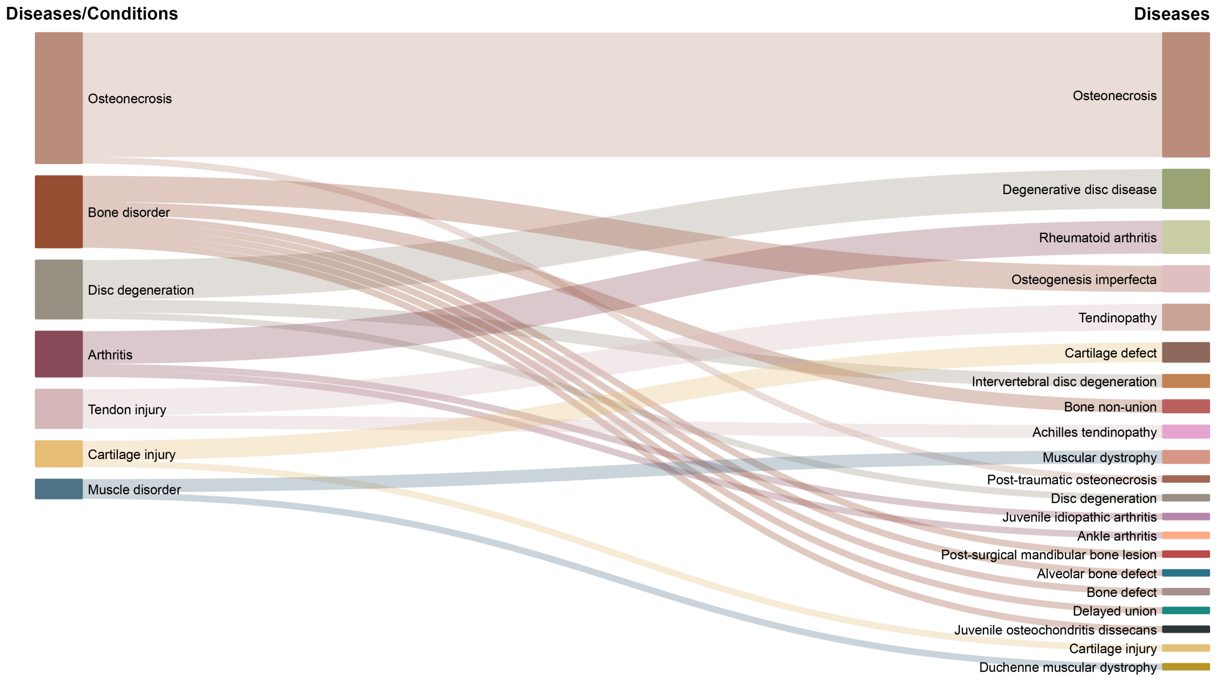

Figure S1. Mapping of Clinical Disease Categories

Sankey diagram illustrating the grouping of clinical disease terms in MSC-based orthopedic/musculoskeletal therapies. The diagram shows how similar disease terms were consolidated into broader diagnostic categories to facilitate the analysis of therapeutic trends across included clinical studies.

3. Translational Mapping of Animal Models, Targeted Organs, and Disease Indications

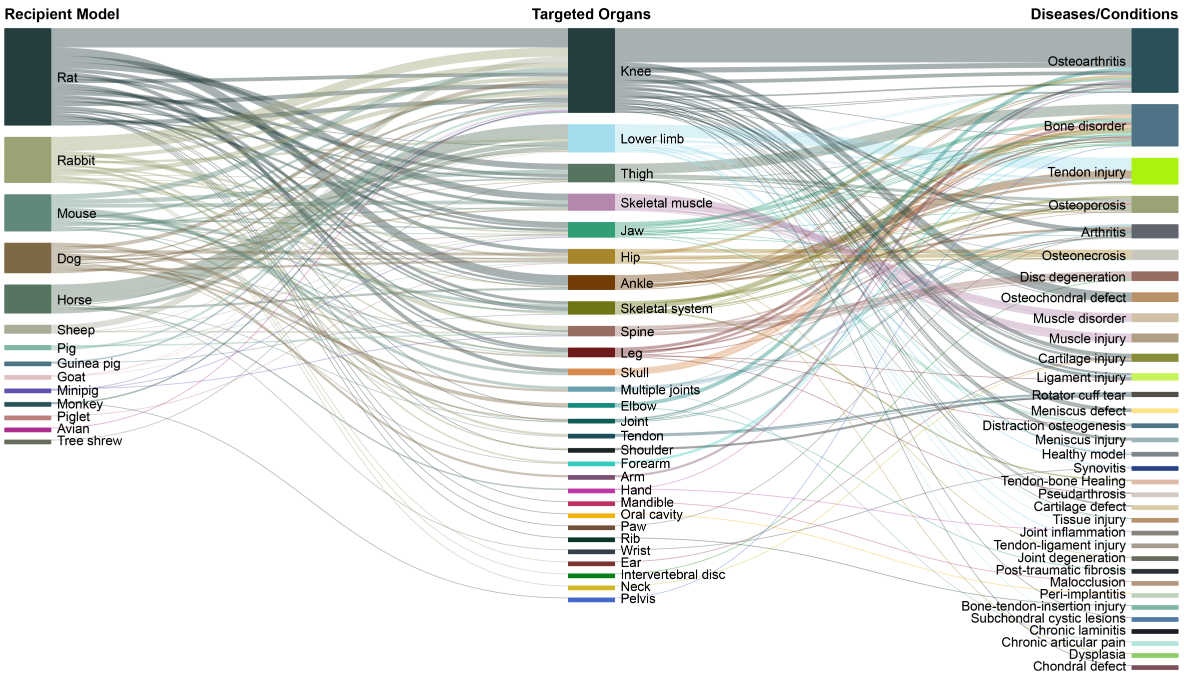

Figure S2. Mapping of Preclinical Models and Disease Targets

Sankey diagram showing the distribution of recipient animal models, targeted organs, and associated diseases/conditions in preclinical studies of stem cell-based therapies. The left column indicates animal models used (e.g., rat, rabbit, mouse, or horse), the center column displays the specific anatomical organs targeted (e.g., knee, lower limb, or thigh), and the right column lists the corresponding orthopedic/musculoskeletal diseases treated (e.g., osteoarthritis, bone disorder, or tendon injury). The connecting lines visualize how different models are applied across target sites and pathological conditions to evaluate the therapeutic effects of stem cells *in vivo*.

4. Mapping Cross-Species Translational Pathways: From Donor to Recipient in Animal Models

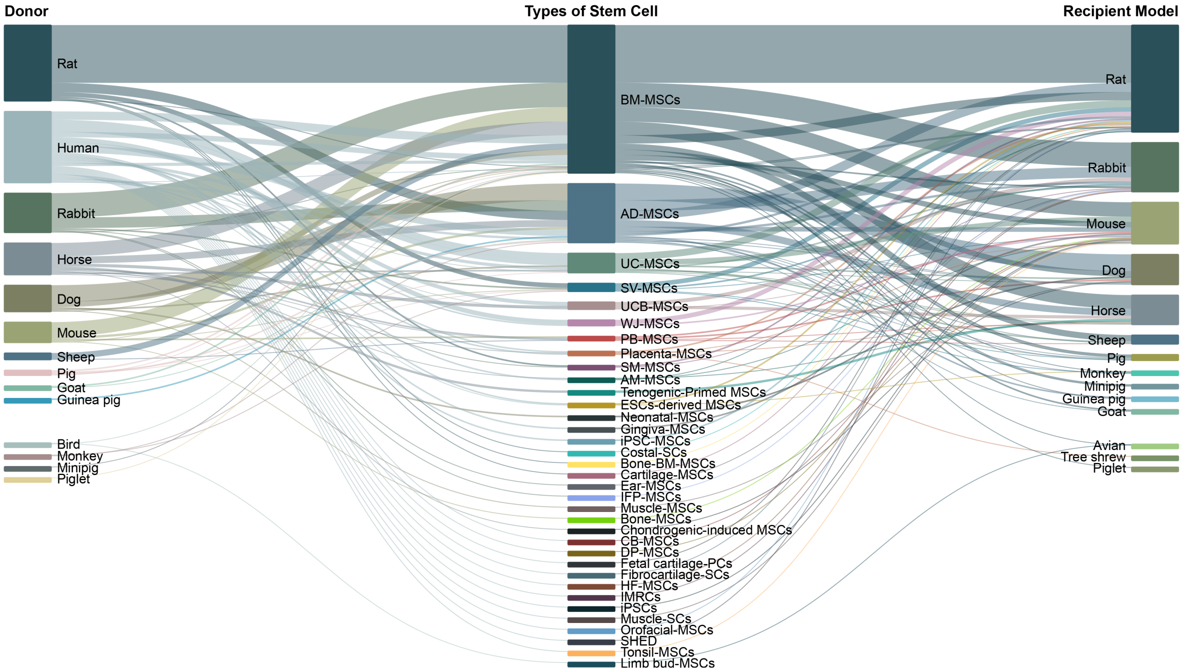

Abbreviations: *AD-MSCs, adipose-derived mesenchymal stem cells; AM-MSCs, amniotic membrane-derived mesenchymal stem cells; BM-MSCs, bone marrow-derived mesenchymal stem cells; Bone-BM-MSCs, bone-derived bone marrow mesenchymal stem cells; CB-MSCs, cord blood-derived mesenchymal stem cells; Costal-SCs, costal cartilage-derived stem cells; DP-MSCs, dental pulp-derived mesenchymal stem cells; ESCs-derived MSCs, embryonic stem cell-derived mesenchymal stem cells; Fetal cartilage-PCs, fetal cartilage-derived progenitor cells; Fibrocartilage-SCs, fibrocartilage-derived stem cells; HF-MSCs, hair follicle-derived mesenchymal stem cells; IFP-MSCs, infrapatellar fat pad-derived mesenchymal stem cells; IMRCs, immunity- and matrix-regulatory cells; iPSC-MSCs, induced pluripotent stem cell-derived mesenchymal stem cells; iPSCs, induced pluripotent stem cells; Limb bud-MSCs, limb bud-derived mesenchymal stem cells; Muscle-SCs, muscle-derived stem cells; PB-MSCs, peripheral blood-derived mesenchymal stem cells; SHED, stem cells from human exfoliated deciduous teeth; SM-MSCs, synovial membrane-derived mesenchymal stem cells; SV-MSCs, synovial-derived mesenchymal stem cells; UCB-MSCs, umbilical cord blood-derived mesenchymal stem cells; UC-MSCs, umbilical cord-derived mesenchymal stem cells; WJ-MSCs, Wharton's jelly-derived mesenchymal stem cells.*

Figure S3. Distribution of Donor Species and Recipient Models

Sankey diagram visualizing the relationships between donor species, types of stem cells, and recipient models in preclinical orthopedic/musculoskeletal studies. The left column identifies stem cell donor species (e.g., rat, human, or rabbit), the middle column lists stem cell types (e.g., BM-MSCs, AD-MSCs, or UC-MSCs), and the right column shows the recipient animal models used in translational research (e.g., rat, rabbit, or mouse). Flow lines demonstrate the origin-to-application trajectory of stem cells, showing common donor-recipient pairings and the diversity of MSC types utilized in regenerative medicine studies.

5. Temporal Trends of Stem Cell Types Used in Preclinical Orthopedic or Musculoskeletal Regeneration

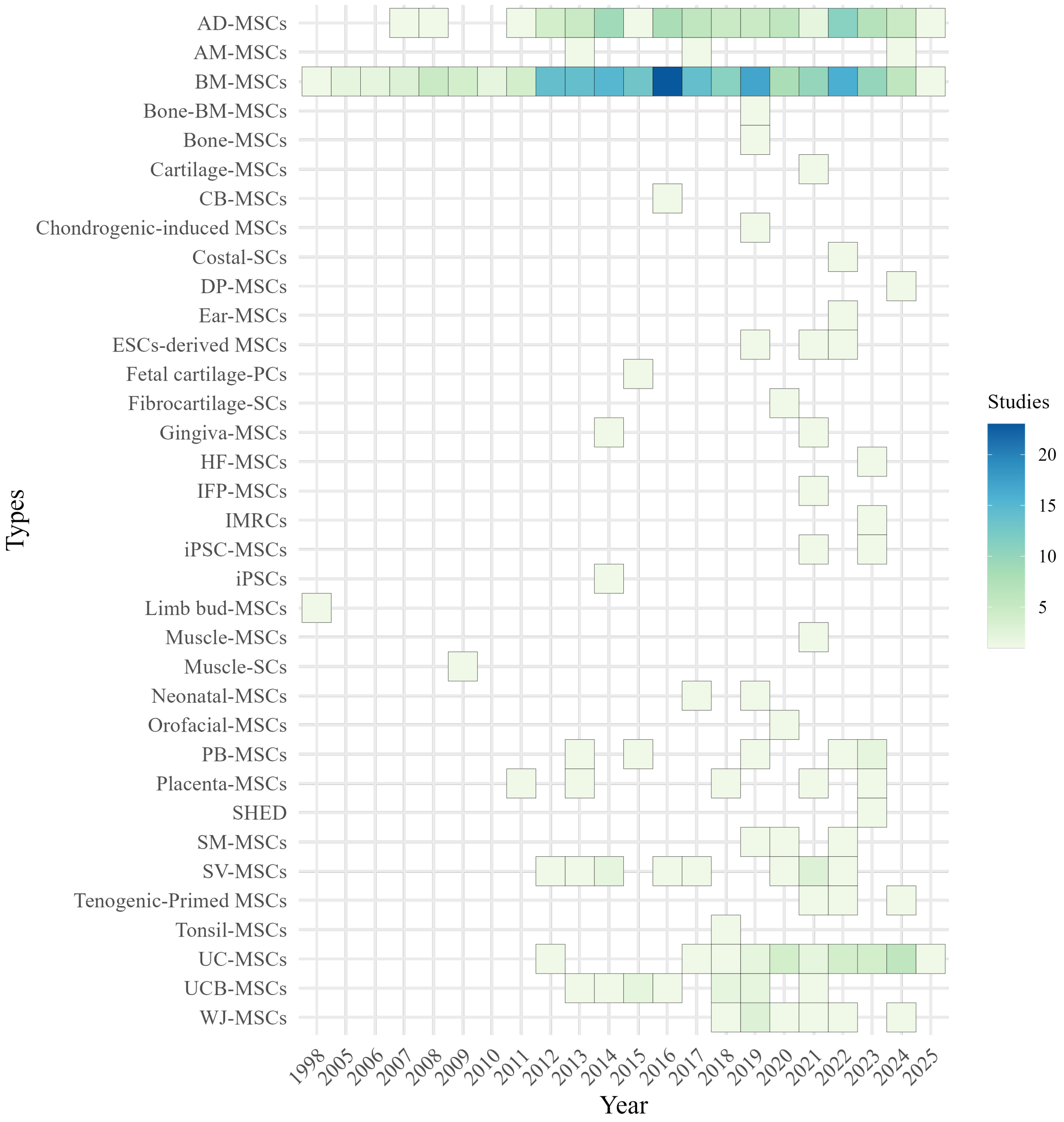

__Abbreviations:__ AD-MSCs, adipose-derived mesenchymal stem cells; AM-MSCs, amniotic membrane-derived mesenchymal stem cells; BM-MSCs, bone marrow-derived mesenchymal stem cells; Bone-BM-MSCs, bone-derived bone marrow mesenchymal stem cells; CB-MSCs, cord blood-derived mesenchymal stem cells; Costal-SCs, costal cartilage-derived stem cells; DP-MSCs, dental pulp-derived mesenchymal stem cells; ESCs-derived MSCs, embryonic stem cell-derived mesenchymal stem cells; Fetal cartilage-PCs, fetal cartilage-derived progenitor cells; Fibrocartilage-SCs, fibrocartilage-derived stem cells; HF-MSCs, hair follicle-derived mesenchymal stem cells; IFP-MSCs, infrapatellar fat pad-derived mesenchymal stem cells; IMRCs, immunity- and matrix-regulatory cells; iPSC-MSCs, induced pluripotent stem cell-derived mesenchymal stem

cells; iPSCs, induced pluripotent stem cells; Limb bud-MSCs, limb bud-derived mesenchymal stem cells; Muscle-SCs, muscle-derived stem cells; PB-MSCs, peripheral blood-derived mesenchymal stem cells; SHED, stem cells from human exfoliated deciduous teeth; SM-MSCs, synovial membrane-derived mesenchymal stem cells; SV-MSCs, synovial-derived mesenchymal stem cells; UCB-MSCs, umbilical cord blood-derived mesenchymal stem cells; UC-MSCs, umbilical cord-derived mesenchymal stem cells; WJ-MSCs, Wharton's jelly-derived mesenchymal stem cells.

Figure S4. Temporal Trends of Preclinical Stem Cell Types

Temporal heatmap of stem cell types used in preclinical orthopedic/musculoskeletal studies (1998-2025). Each row represents a distinct stem cell source, and each column a year. The color intensity corresponds to the annual publication frequency, with darker shades indicating a higher number of studies. The data highlights the sustained predominance of BM-MSCs, the growing prominence of AD-MSCs, and the recent emergence of perinatal tissue-derived MSCs (placenta-MSC, UC-MSCs, UCB-MSCs, and WJ-MSCs).

6. Mapping Stem Cell Applications in Orthopedic or Musculoskeletal Regeneration: A Sankey Diagram Analysis

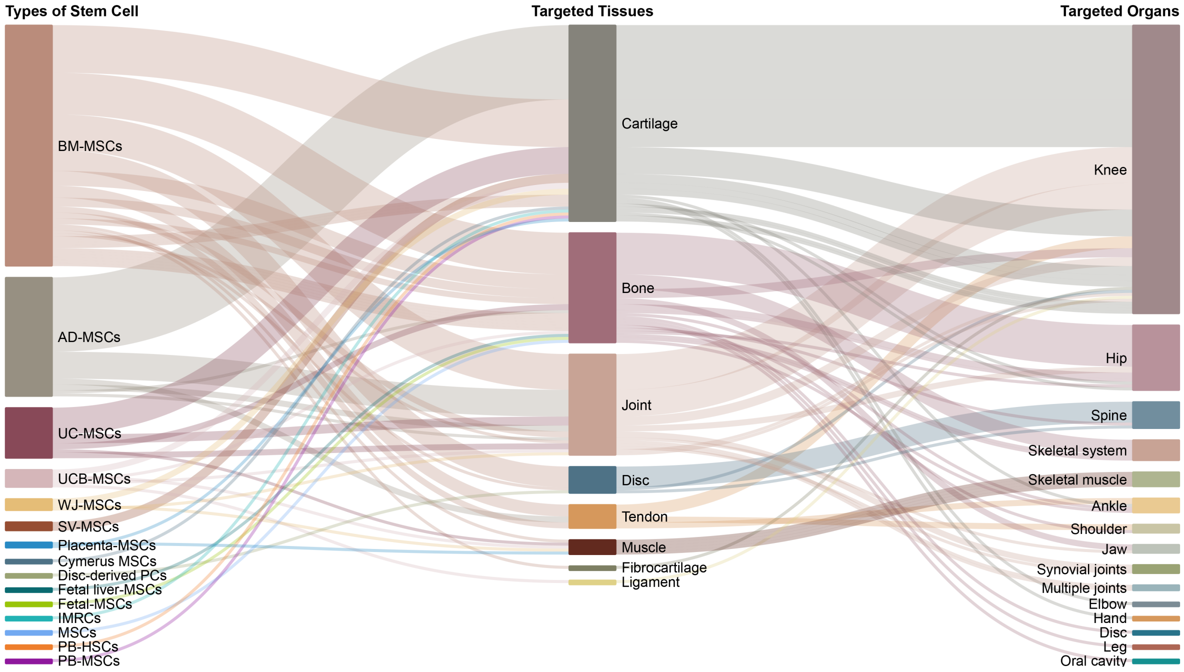

Abbreviations: *BM-MSCs, bone marrow-derived mesenchymal stem cells; AD-MSCs, adipose-derived mesenchymal stem cells; UC-MSCs, umbilical cord-derived mesenchymal stem cells; UCB-MSCs, umbilical cord blood-derived mesenchymal stem cells; WJ-MSCs, Wharton's jelly-derived mesenchymal stem cells; SV-MSCs, synovial-derived mesenchymal stem cells; Placenta-MSCs, placenta-derived mesenchymal stem cells; Disc-derived PCs, progenitor cells; IMRCs, immunity- and matrix-regulatory cells; MSCs, mesenchymal stem cells; PB-HSCs, peripheral blood-derived hematopoietic stem cells; PB-MSCs, peripheral blood-derived mesenchymal stem cells.*

Figure S5. Stem Cell Pathways Across Tissues and Organs

Sankey diagram illustrating the relationship between stem cell types, targeted tissue, and anatomical organs in orthopedic/musculoskeletal regenerative therapies. The diagram shows how various stem cell sources are applied across musculoskeletal tissues and specific organ sites, showing the most frequently studied therapeutic pathways

7. Temporal Trends in the Clinical Use of Different Stem Cell Types

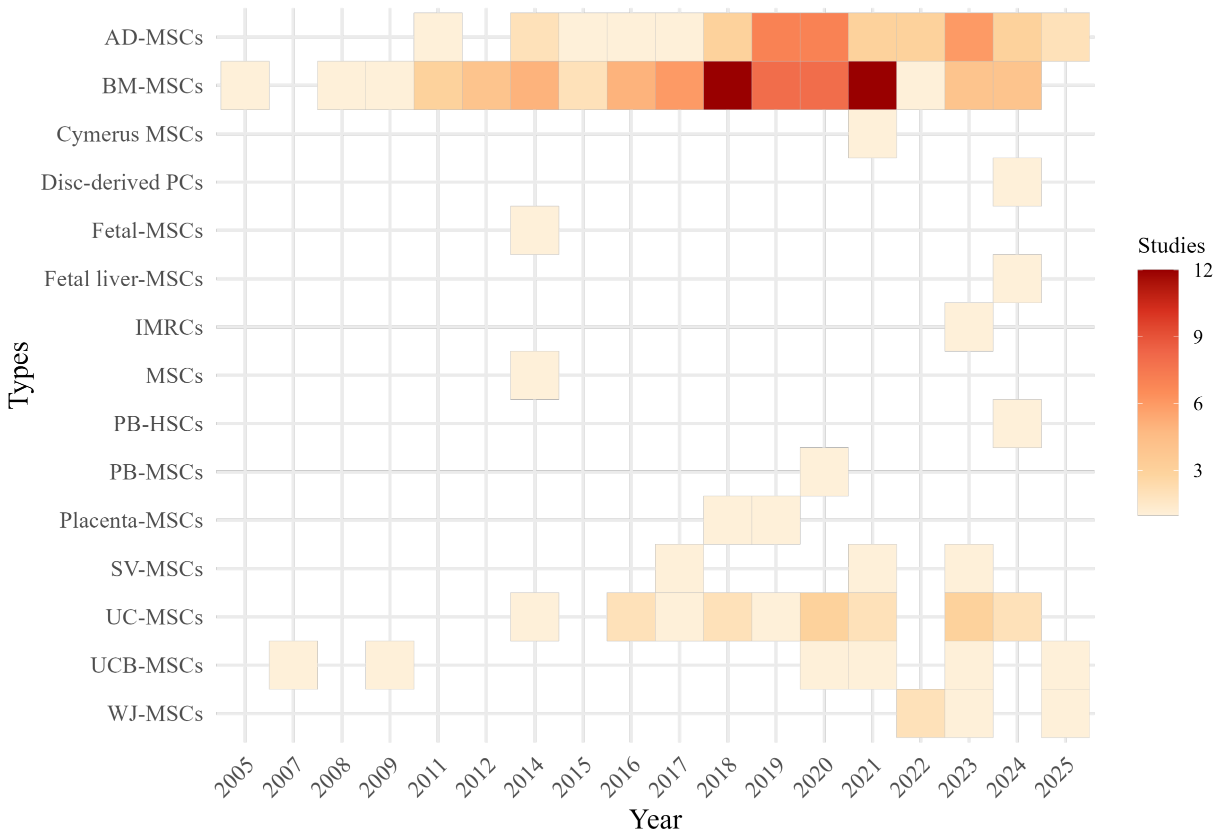

Abbreviations: *BM-MSCs, bone marrow-derived mesenchymal stem cells; AD-MSCs, adipose-derived mesenchymal stem cells; UC-MSCs, umbilical cord-derived mesenchymal stem cells; UCB-MSCs, umbilical cord blood-derived mesenchymal stem cells; WJ-MSCs, Wharton's jelly-derived mesenchymal stem cells; SV-MSCs, synovial-derived mesenchymal stem cells; Placenta-MSCs, placenta-derived mesenchymal stem cells; Disc-derived PCs, progenitor cells; IMRCs, immunity- and matrix-regulatory cells; MSCs, mesenchymal stem cells; PB-HSCs, peripheral blood-derived hematopoietic stem cells; PB-MSCs, peripheral blood-derived mesenchymal stem cells.*

Figure S6. Temporal Trends of Clinical Stem Cell Types

Heatmap showing the annual distribution of stem cell types used in clinical studies from 2005 through early 2025. The color gradient indicates the frequency of each stem cell type per year. The heatmap highlights the foundational role of BM-MSCs and the significant rise of AD-MSCs and perinatal sources in recent years.
